# Evaluation of Patient-Centered Outcomes Associated With the Acceleration of Upper Incisor Decrowding Using Self-Ligating Brackets With or Without Piezocision in Comparison With Traditional Brackets: A Three-Arm Randomized Controlled Trial

**DOI:** 10.7759/cureus.26467

**Published:** 2022-06-30

**Authors:** Heba M Al-Ibrahim, Mohammad Y Hajeer, Ahmad S Burhan, Issam Alkhouri, Youssef Latifeh

**Affiliations:** 1 Department of Orthodontics, University of Damascus Faculty of Dentistry, Damascus, SYR; 2 Department of Oral and Maxillofacial Surgery, University of Damascus Faculty of Dentistry, Damascus, SYR; 3 Department of Internal Medicine, University of Damascus Faculty of Medicine, Damascus, SYR

**Keywords:** alignment, severe crowding, piezocision, self-ligating brackets, discomfort, pain

## Abstract

Introduction

The pain and discomfort associated with orthodontic treatment are considered undesirable complications which may negatively affect the patient’s cooperation and reduce the efficiency of orthodontic treatment. This trial aimed to assess patient-centered outcomes in the treatment of class I malocclusion with severe crowding using self-ligating brackets (SLBs) with or without an acceleration procedure (piezocision) compared to the traditional brackets (TBs) in a three-arm trial.

Materials and Methods

Sixty-six patients (51 females, 15 males; mean age ± SD: 20.08 ± 2.61 years) attending the Department of Orthodontics, the University of Damascus, Damascus (Syria) from April 2019 to October 2020 participated in this study. The patients were randomly allocated to three groups: the TBs s group (n = 22; mean age ± SD: 18.72 ± 2.42), the SLBs group (n = 22; mean age ± SD: 20.48 ± 2.84), and the SLBs with the piezocision group (SLBs+P; n = 22; mean age ± SD: 19.17 ± 2.59). Patient-centered outcomes were assessed using two standardized questionnaires depending on visual analog scales (VAS) for the majority of the questions and a binary scale (Yes/No) for the last two questions in the second questionnaire. The levels of pain, discomfort, swelling, difficulties with mastication, swallowing, and jaw movement restriction were measured at five assessment times: one day (T1), 3 days (T2), 7 days (T3), 14 days (T4), and 28 days (T5) after the beginning of treatment. The satisfaction levels, acceptance to undergo the applied treatment again, and willingness to advise a friend to receive similar treatment were measured at the last assessment time (T5). One-way ANOVA test or its alternative nonparametric test (i.e., Kruskal-Wallis test) was utilized to compare the three groups.

Results

There were statistically significant differences between the three groups regarding pain, discomfort, swelling, difficulties with mastication, problems with swallowing, and jaw movement restriction during the first three assessment times only (T1, T2, and T3; P < 0.001). The differences were mainly between the SLBs+P group and the other two groups, where the mean values were greater in the SLBs+P group. Otherwise, there were no significant statistical differences between the SLBs and the TBs groups. Concerning patients’ satisfaction with the provided treatment, a statistically significant difference between the three groups was detected after 28 days (T5; P < 0.001). The SLBs+P group showed the lowest mean values, whereas there were no significant differences between the two other groups.

Conclusion

The levels of pain and discomfort, swelling, difficulties in mastication and swallowing, and restriction of jaw movement were greater in SLBs with the piezocision group compared to the sole use of SLBs or TBs in the first week only. The patients showed a high level of satisfaction with the applied therapeutic procedures, which means that SLBs alone or in combination with piezocision can be an accepted treatment modality by patients in the acceleration of orthodontic tooth movement.

## Introduction

Severe crowding is considered one of the most important orthodontic problems, which negatively affects patients’ psychological states [1,2]. The importance of treatment in these cases comes from the necessity of improving the cosmetic aspects and maintaining the periodontal status and oral health by providing good teeth alignment. The length of the treatment period and the consequential problems such as dental caries and root resorption have led many patients, especially adults, to refuse to receive any orthodontic treatment [1,2]. Therefore, many researchers have applied various acceleration methods in an attempt to reduce treatment time [3-5]. A wide range of procedures is used to quicken the orthodontic dental movement, such as physical, mechanical, and surgical [1,6,7]. Although many previous studies have proven the effectiveness of surgical interventions in accelerating dental movement, some of them have shown that these interventions have been accompanied by different levels of pain and discomfort, especially the invasive surgical methods requiring flaps elevation [8]. Contradictorily, some researchers have claimed that minimally invasive surgical interventions, such as flapless corticotomy, cause less pain making them more tolerable [9-11].

The orthodontic treatment causes varying levels of pain and discomfort, resulting in reduced cooperation and commitment of patients [12,13]. Compression of the periodontal ligaments following the application of orthodontic forces, and sometimes greater chewing forces than normal levels, are considered the leading causes of the resulting pain [12,13]. Regarding the influence of factors related to the brackets system used, especially in the leveling and alignment phase, the pain perception is proportional to the magnitude of forces applied to the teeth [11]. Friction between the elements of the fixed orthodontic appliance (i.e., brackets and wires) plays an essential role in the amount of orthodontic movement [3,4]. The friction amount varies according to the physical properties of the wire and the brackets materials, the dimensions of the applied wires, and the ligation type used [12,13]. Moreover, it has been shown in many previous studies that other factors play a role in pain perception, such as the patient’s age and the severity of the malocclusion [3,4].

Several studies have evaluated pain and discomfort and functional impairments associated with orthodontic treatment of crowding cases with or without extraction [14-16]. The results differ from one study to another according to many factors, such as the severity of malocclusion and the applied brackets system (self-ligating or traditional brackets (TBs)). Some studies have shown that the pain levels were less in the self-ligating brackets (SLBs) group than in the TBs group [17,18]. On the contrary, some have reported that the pain associated with the use of SLBs was greater [19,20]. While other studies have revealed that there was no effect of the brackets system used on the pain and discomfort associated with orthodontic treatment [21-25]. Regarding the participation of SLBs with surgical acceleration methods, only one study by Charavet et al. evaluated the outcomes related to patients associated with this type of therapeutic intervention [9]. Charavet et al. conducted their study on a sample of adult patients who suffered from mild to moderate crowding on the two jaws and found no difference in pain perception between the experimental group (SLBs + piezocision) and the control group [9].

As a consequence of the discrepancy in the results obtained from previous studies and due to the lack of evidence in the medical literature concerning the combination of SLBs with piezocision in treating severe crowding cases with extraction, this randomized controlled clinical trial was conducted to assess the levels of pain, discomfort, and functional impairments associated with the treatment of severe anterior maxillary crowding using SLBs combined with piezocision compared to SLBs alone or TBs alone in adult patients. The null hypothesis employed in the current trial was that there were no differences in the patient-centered outcomes between the three studied groups.

## Materials and methods

Study design and registration

The principal researcher examined patients attending the Department of Orthodontics at Damascus University, Dental School, between April 2019 and October 2020. This trial was conducted based on the guidelines of CONsolidated Standards of Reporting Trials (CONSORT), where all procedures were performed as were priorly scheduled [26]. The present study was recorded at ClinicalTrials.gov (ID: NCT05416242) retrospectively, i.e., the registration was done after the onset of this trial. The Local Research Ethics Committee Approval was acquired from the University of Damascus (UDDS-567-2019GD/SRC-58879), and the funding for this trial was received from the Postgraduate Research Budget at Damascus University (Ref no: 34235656781JHF).

Sample size calculation

The sample size was calculated using Minitab® Version 18 (Minitab Inc., State College, Pennsylvania, USA). It was assumed that the least significant difference in the pain level was 10 mm on the visual analog scale (VAS) with a standard deviation of 9.4 mm, according to Jahanbin et al.’s study [27]. Using an ANOVA test with a power of 85% and a significance level of 5%, 21 participants were needed in each group. One participant was added to avoid any potential attrition, resulting in 22 participants in each group.

Patients’ recruitment and eligibility criteria

**Figure 1:**
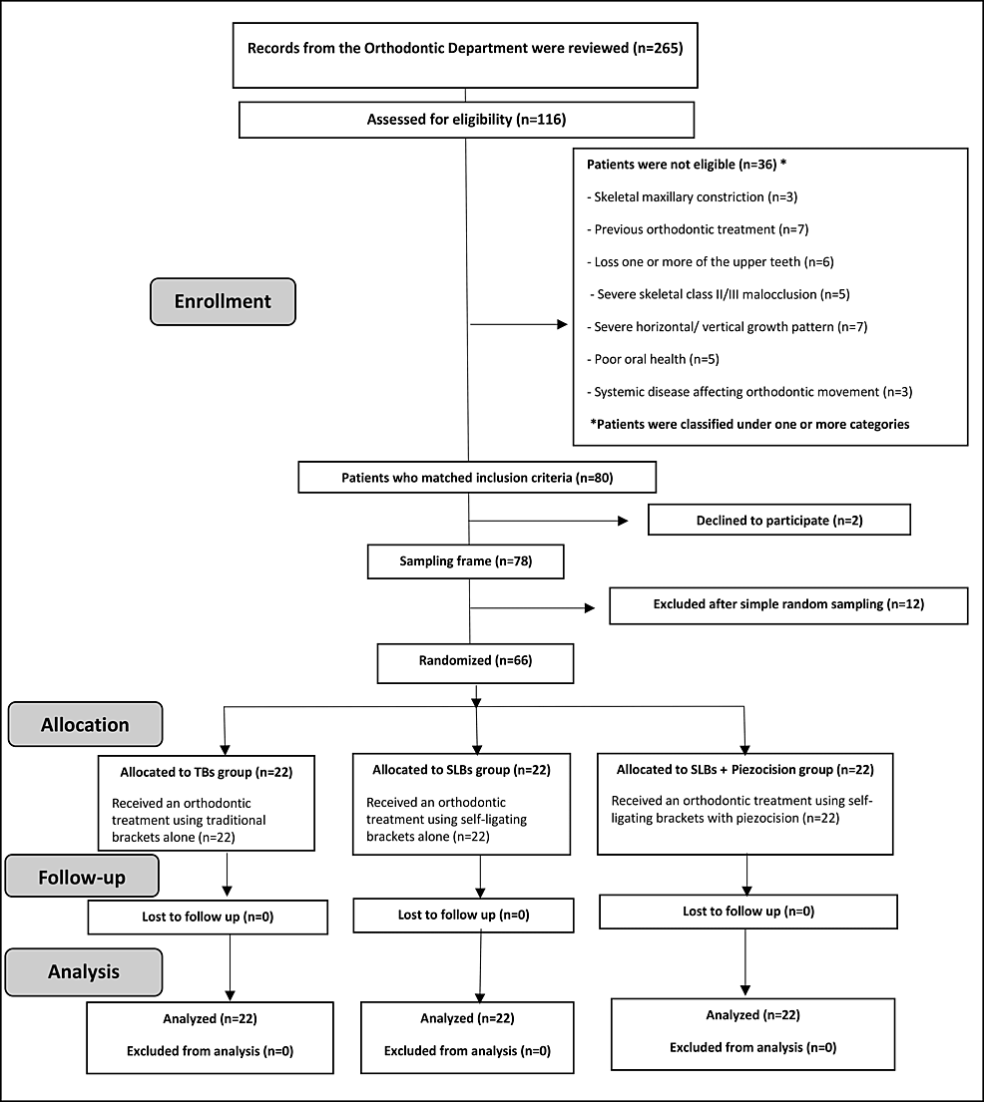
Flow diagram of patients’ recruitment, follow-up, and entry to data analysis. SLB: self-ligating bracket; TB: traditional bracket

After clinical examination of 116 patients at the Department of Orthodontics, the University of Damascus, it was found that 80 individuals matched the selection criteria. All patients received sufficient explanation about the orthodontic and surgical procedures in this research, and then out of 78 participants who agreed to take part in this study, 66 were randomly selected (Figure 1). Information sheets were given to all selected patients; then, informed consent forms were acquired. The inclusion criteria were: (1) The patient is between 18 and 28 years old, (2) maxillary severe crowding (> 6 mm) demanding extraction of the first premolars, (3) Little’s Index is greater than 7 mm, (4) 0-4 mm overbite, (5) normal maxillary incisors inclination, (6) no missing teeth. The exclusion criteria were: (1) any disease impacting orthodontic movement, (2) any congenital syndromes or clefts, (3) inadequate oral health, and (4) skeletal maxillary constriction.

Randomization, allocation concealment, and blinding

SPSS® Version 20 (SPSS for Windows, version 20, IBM Corporation, Australia) was the chosen software to produce patient allocation between the three groups with an allocation ratio of 1:1:1. The allocation concealment was applied using a series of random numbers; then, the allocation sequence was masked using numbered, wrapped, and sealed envelopes, which were not allowed to open before premolars extraction. An academic person not contributory to this study was responsible for conducting the random allocation sequence. The 66 patients were divided randomly into three groups: The first group underwent orthodontic treatment using TBs alone (TBs group); the second group underwent orthodontic treatment using SLBs alone (SLBs group), and the third group underwent orthodontic treatment using SLBs with piezocision (SLBs+P). Blinding was limited to the data analysis only, and it was not applicable to either participants or practitioners.

Orthodontic procedures

Orthodontic treatment using active SLBs (Empower®, American Orthodontics, Sheboygan, Wisconsin, USA) or TBs (Master Series®, American Orthodontics, Sheboygan, Wisconsin, USA), with a 0.022-inch slot high and MBT prescription were used. The brackets system was chosen for each patient according to his/her group, then the brackets were bonded after 7 days of the first upper premolars extraction. In the three groups, the archwire sequence was as follows: 0.012-inch Nitinol (NiTi), 0.014-inch NiTi, 0.016-inch NiTi, 0.016×0.022-inch NiTi, 0.017×0.025-inch NiTi, and finally 0.017×0.025-inch stainless steel (SS) (American Orthodontics, Sheboygan, Wisconsin, USA) [10]. Replacing wires was accomplished when the used wire became neutral with the ability to insert the next wire without applying exaggerated force. When the little’s index became less than 1 mm, it represented the end of the leveling and alignment phase [10].

Piezocision surgical procedure

Initially, it was taken a digital periapical radiograph to precisely locate the incisions away from teeth roots. The patient was requested to rinse with 0.12% chlorhexidine gluconate (Oral-B, Procter & Gamble Company, USA) before applying the surgical intervention. The surgical intervention was accomplished, where 3 mm-deep and 5 to 8 mm-long incisions were conducted using a Piezosurgical micro-saw with a BS1 cutting tip (Implant Center™ 2, Satelec, France). Patients were asked to follow a soft diet for 3 days after the surgical intervention and adhere to the mouth rinse for 10 days [10]. It was not allowed to use any anti-inflammatory drugs, but in the case of moderate or severe pain, the patient could take one to two tablets of paracetamol 500 mg, provided filling out the questionnaires first.

Outcome measures: pain, discomfort, and functional impairments

**Figure 2:**
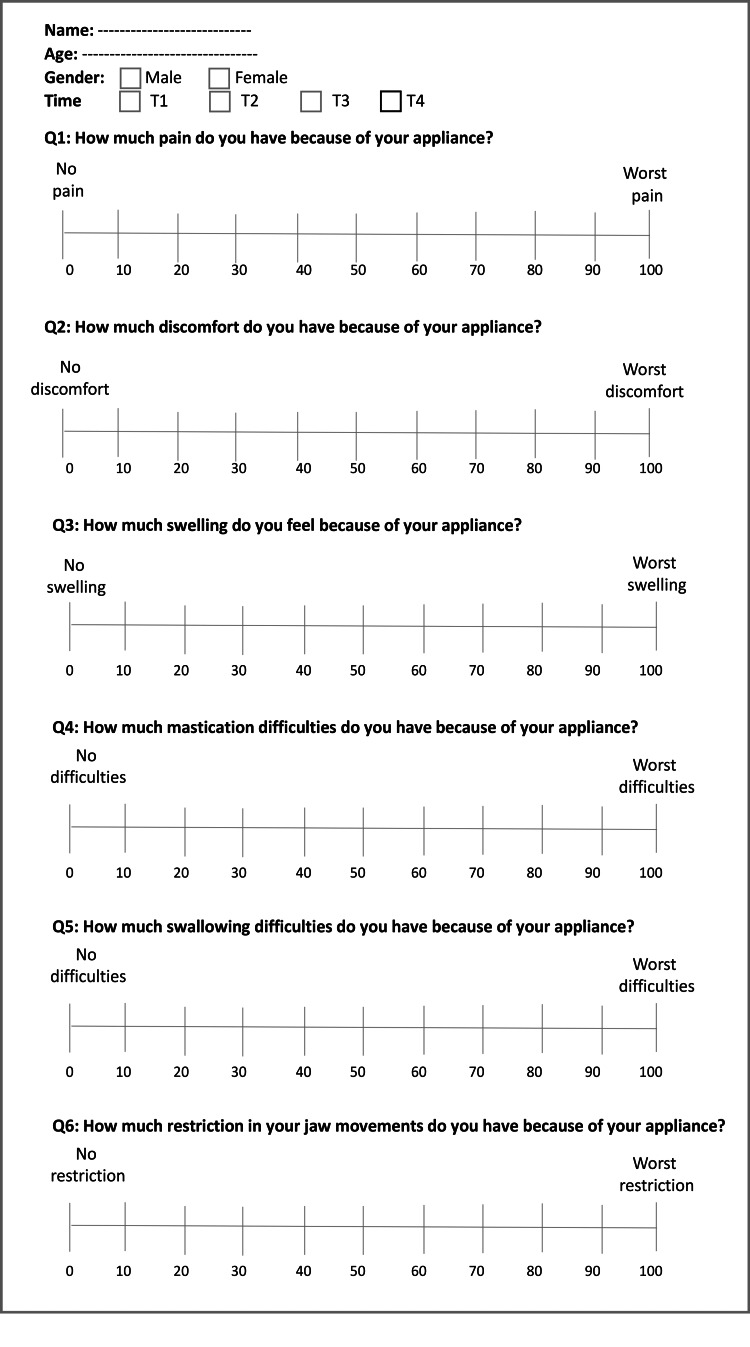
The first questionnaire was provided on 1 day, 3 days, 7 days, and 14 days after the beginning of treatment.

**Figure 3:**
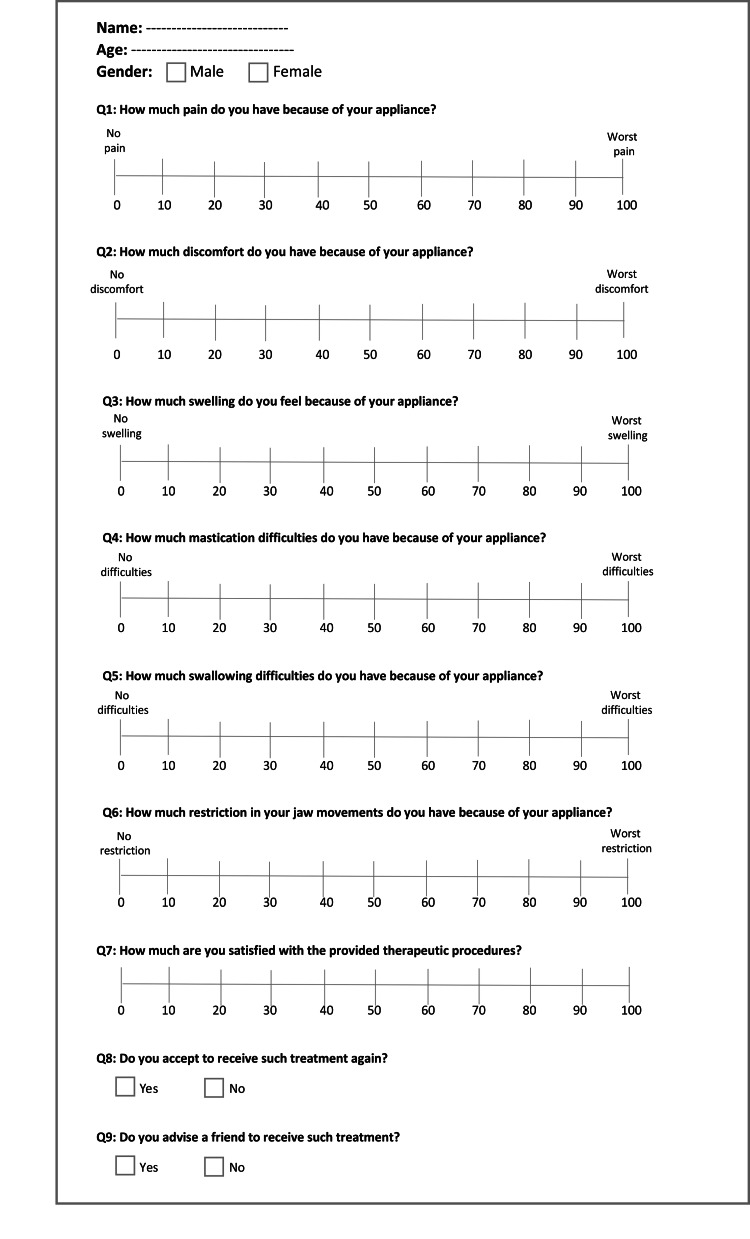
The second questionnaire was provided 28 days following the beginning of treatment.

The outcome measures contained the levels of pain, discomfort, and functional impairments within 28 days after the onset of the orthodontic treatment using two questionnaires. The first questionnaire included six questions: (i) the levels of pain that patient sensed; (ii) the levels of discomfort that patient sensed; (iii) the levels of swelling that patient sensed; (iv) the level of mastication difficulties that patients sensed; (v) the levels of swallowing difficulties that patient sensed, and (vi) the levels of jaw movement restriction that patients sensed. The second questionnaire involved the same questions included in the first questionnaire, in addition to three questions: (vii) the levels of satisfaction, (viii) acceptance to undergo the applied treatment again, and (ix) advising a friend to receive the same treatment. The first questionnaire was answered at four assessment times: one day, 3 days, 7 days, and 14 days after the beginning of treatment (Figure 2), while the second questionnaire was answered after 28 days (Figure 3). The VAS was used for all questions except for the last two ones, where a two-point scale (Yes/No) was used. Each patient had to determine a point expressed his/her perceived levels of each studied variable on the VAS, a line of 100-mm length.

Statistical analysis

SPSS® Version 20 (SPSS for Windows, version 20, IBM Corporation, Australia) was the chosen software for statistical analysis. The Shapiro-Wilk test was used to distinguish the normal distribution of data. One-way ANOVA test or its alternative nonparametric test (i.e., Kruskal-Wallis test) was utilized to make a comparison between the three groups. For pairwise comparisons, the posthoc Bonferroni test or its alternative nonparametric test (i.e., Mann-Whitney test) was applied. The results of all tests were significant at p ≤ 0.05, except for the Mann-Whitney test results, which were significant at p ≤ 0.017, where the significance level was modified according to Bonferroni correction.

## Results

Sixty-six patients (51 females, 15 males; mean age ± SD: 20.08 ± 2.61 years) participated in this trial. Table 1 shows the baseline characteristics of the participants in the three groups. There was no patient withdrawal from the study; consequently, all 66 patients were included in the data analysis.

**Table 1:** Basic sample characteristics (gender and age). n: number of patients; SD: standard deviation; Min.: minimum; Max.: maximum; TBs: traditional brackets; SLBs: self-ligating brackets; SLBs+P: self-ligating brackets + piezocision *employing chi-square test †employing One-way ANOVA

Group	N	Gender	N (%)	P-value^*^	Mean age (SD)	Min. age	Max. age	P-value†
TBs	22	Male	6 (27.27 %)	0.568	18.72 (2.42)	17.00	24.10	0.647
		Female	16 (72.72 %)					
SLBs	22	Male	5 (22.72 %)		20.48 (2.84)	17.10	24.50	
		Female	17 (77.27 %)					
SLBs+P	22	Male	4 (18.18 %)		19.17 (2.59)	17.20	23.80	
		Female	18 (81.81 %)					
All sample	66	Male	15 (22.72 %)		20.08 (2.61)	17.00	24.50	
		Female	51 (77.27 %)					

There were statistically significant differences between the three groups regarding pain, discomfort, swelling, difficulties with mastication, problems with swallowing, and jaw movement restriction during the first three assessment times (P < 0.001, Tables 2-4). In contrast, there were no statistically significant differences between the three groups regarding the previously mentioned variables at the last two assessment times (P > 0.05; Table 5).

**Table 2:** Descriptive statistics of patient-centered variables of questions 1 and 2 at five assessment times in the three groups using visual analog scales and the results of the significance testing. SD: standard deviation; Min: minimum; Max: maximum; CI: confidence interval; T1: after 24 hours of the beginning of orthodontic treatment; T2: after 4 days; T3: after 7 days; T4: after 14 days; T5: after 28 days; TBs: traditional brackets; SLBs: self-ligating brackets; SLBs+P: self-ligating brackets + piezocision; NS: There was no statistically significant difference at P > 0.05. †Employing one-way ANOVA test or Kruskal-Wallis test *There was a statistically significant difference at P < 0.001 **There was a statistically significant difference at P < 0.01

	Group	Mean	SD	Min	Max	95% CI for difference		P-value†	Significance
						Lower bound	Upper bound		
Q1: Pain									
T1	TBs	31.85	6.64	20	44	28.74	34.96	>0.001	*
	SLBs	30.42	6.98	15	40	27.05	33.79		
	SLBs+P	50.58	8.75	35	67	46.36	54.80		
T2	TBs	7.95	5.82	0	20	5.22	10.68	0.002	**
	SLBs	7.26	3.60	2	15	5.53	9.00		
	SLBs+P	15.32	10.84	0	37	10.09	20.54		
T3	TBs	2.90	1.86	0	6	2.03	3.77	>0.001	*
	SLBs	3.11	1.62	1	6	2.32	3.89		
	SLBs+P	12.79	8.82	0	6	8.53	17.04		
T4	TBs	0.70	0.86	0	2	0.30	1.10	0.976	NS
	SLBs	0.74	0.87	0	2	0.32	1.16		
	SLBs+P	0.89	1.28	0	5	0.27	1.51		
T5	TBs	0.30	0.65	0	2	-0.01	0.61	0.873	NS
	SLBs	0.26	0.65	0	2	-0.05	0.58		
	SLBs+P	0.68	1.60	0	2	-0.09	1.46		
Q2: Discomfort									
T1	TBs	33.90	8.27	20	45	30.03	37.77	>0.001	*
	SLBs	32.63	6.47	20	42	29.51	35.75		
	SLBs+P	51.42	6.85	38	63	48.12	54.73		
T2	TBs	18.05	2.16	1	33	13.52	22.58	>0.001	*
	SLBs	8.37	3.98	2	15	6.45	29		
	SLBs+P	22.00	14.22	1	54	15.14	28.86		
T3	TBs	10.50	6.17	1	22	7.61	13.39	>0.001	*
	SLBs	2.47	1.26	1	5	1.86	3.08		
	SLBs+P	16.53	7.82	1	17	12.76	20.30		
T4	TBs	0.95	1.46	0	5	0.26	1.64	0.944	NS
	SLBs	1.00	1.49	0	5	0.28	1.72		
	SLBs+P	1.21	1.75	0	5	0.37	2.05		
T5	TBs	0.60	0.75	0	2	0.25	0.95	0.904	NS
	SLBs	0.63	0.83	0	2	0.23	1.03		
	SLBs+P	0.53	0.77	0	2	0.15	0.90		

**Table 3:** Descriptive statistics of patient-centered variables of questions 3 and 4 at five assessment times in the three groups using visual analog scales and the results of the significance testing. SD: standard deviation; Min: minimum; Max: maximum; CI: confidence interval; T1: after 24 hours of the beginning of orthodontic treatment; T2: after 4 days; T3: after 7 days; T4: after 14 days; T5: after 28 days; TBs: traditional brackets; SLBs: self-ligating brackets; SLBs+P: self-ligating brackets+ piezocision; NS: There was no statistically significant difference at P > 0.05 †Employing a one-way ANOVA test or Kruskal-Wallis test *There was a statistically significant difference at P < 0.001

	Group	Mean	SD	Min	Max	95% CI for difference		P-value^†^	Significance
						Lower bound	Upper bound		
Q3: Swelling									
T1	TBs	0.05	0.22	0	1	-0.05	0.15	>0.001	*
	SLBs	0.05	0.22	0	1	-0.06	0.16		
	SLBs+P	37.16	8.46	20	48	33.08	41.24		
T2	TBs	0.05	0.22	0	1	-0.05	0.15	>0.001	*
	SLBs	0.05	0.22	0	1	-0.06	0.16		
	SLBs+P	20.74	10.01	4	37	15.91	25.56		
T3	TBs	0.05	0.22	0	1	-0.05	0.15	>0.001	*
	SLBs	0.05	0.22	0	1	-0.06	0.16		
	SLBs+P	0.95	1.12	0	4	0.40	1.49		
T4	TBs	0.05	0.22	0	1	-0.05	0.15	0.160	NS
	SLBs	0.05	0.22	0	1	-0.06	0.16		
	SLBs+P	0.63	0.57	0	1	-0.13	1.39		
T5	TBs	0.05	0.22	0	1	-0.05	0.15	0.752	NS
	SLBs	0.05	0.22	0	1	-0.06	0.16		
	SLBs+P	0.11	0.31	0	1	-0.05	0.26		
Q4: Mastication									
T1	TBs	30.95	6.25	20	39	28.02	33.88	>0.001	*
	SLBs	30.84	6.59	20	40	27.66	34.02		
	SLBs+P	58.95	12.94	13	73	52.71	65.19		
T2	TBs	7.95	4.31	2	18	5.93	9.97	>0.001	*
	SLBs	4.32	2.81	1	12	2.96	5.67		
	SLBs+P	21.79	13.94	2	55	15.07	28.51		
T3	TBs	4.45	2.96	2	11	3.06	5.84	>0.001	*
	SLBs	4.00	3.33	1	11	2.39	5.61		
	SLBs+P	11.74	7.97	1	30	7.89	15.58		
T4	TBs	1.00	1.45	0	5	0.32	1.68	0.840	NS
	SLBs	1.05	1.47	0	5	0.34	1.76		
	SLBs+P	1.32	1.70	0	5	0.50	2.14		
T5	TBs	0.50	0.76	0	2	0.14	0.86	0.722	NS
	SLBs	0.58	0.83	0	2	0.18	0.98		
	SLBs+P	0.84	1.16	0	4	0.28	1.40		

**Table 4:** Descriptive statistics of patient-centered variables of questions 5, 6, and 7 at the assessment times in the three groups using visual analog scales and the results of the significance testing. SD: standard deviation; Min: minimum; Max: maximum; CI: confidence interval; T1: after 24 hours of the beginning of orthodontic treatment; T2: after 4 days; T3: after 7 days; T4: after 14 days; T5: after 28 days; TBs: traditional brackets; SLBs: self-ligating brackets; SLBs+P: self-ligating brackets + piezocision; NS: There was no statistically significant difference at P > 0.05. †Employing a one-way ANOVA test or Kruskal-Wallis test *There was a statistically significant difference at P < 0.001

	Group	Mean	SD	Min	Max	95% CI for difference		P-value^†^	Significance
						Lower bound	Upper bound		
Q5: Swallowing									
T1	TBs	13.25	6.12	10	30	10.38	16.12	>0.001	*
	SLBs	13.16	5.82	10	30	10.35	15.96		
	SLBs+P	25.42	10.08	10	43	20.56	30.28		
T2	TBs	3.70	2.49	1	9	2.53	4.87	>0.001	*
	SLBs	3.53	2.43	1	10	2.35	4.70		
	SLBs+P	12.26	9.31	1	33	7.77	16.75		
T3	TBs	0.05	0.22	0	1	-0.05	0.15	>0.001	*
	SLBs	0.05	0.22	0	1	-0.06	0.16		
	SLBs+P	2.32	2.08	0	6	1.31	3.32		
T4	TBs	0.05	0.22	0	1	-0.05	0.15	0.752	NS
	SLBs	0.05	0.22	0	1	-0.06	0.16		
	SLBs+P	0.11	0.31	0	1	-0.05	0.26		
T5	TBs	0.05	0.22	0	1	-0.05	0.15	0.160	NS
	SLBs	0.05	0.22	0	1	-0.06	0.16		
	SLBs+P	0.42	1.01	0	4	-0.07	0.91		
Q6: Jaw movement restriction									
T1	TBs	1.30	1.86	0	4	0.43	2.17	>0.001	*
	SLBs	0.79	1.35	0	3	0.14	1.44		
	SLBs+P	32.16	10.17	20	53	27.25	37.06		
T2	TBs	0.90	1.25	0	4	0.31	1.49	>0.001	*
	SLBs	0.68	1.05	0	3	0.17	1.19		
	SLBs+P	7.16	4.90	1	16	4.80	9.52		
T3	TBs	0.10	0.44	0	2	-0.11	0.31	>0.001	*
	SLBs	0.05	0.22	0	1	-0.06	0.16		
	SLBs+P	4.26	4.65	0	14	2.02	6.51		
T4	TBs	0.05	0.22	0	1	-0.06	0.16	0.999	NS
	SLBs	0.05	0.22	0	1	-0.05	0.26		
	SLBs+P	0.05	0.22	0	1	-0.06	0.16		
T5	TBs	0.05	0.22	0	1	-0.05	0.15	0.999	NS
	SLBs	0.05	0.22	0	1	-0.06	0.16		
	SLBs+P	0.05	0.22	0	1	-0.06	0.16		
Q7: Satisfaction									
T5	TBs	98.30	3.24	90	100	96.78	99.82	>0.001	*
	SLBs	98.95	2.22	92	100	97.88	100.02		
	SLBs+P	91.89	7.79	79	100	88.14	95.65		

**Table 5:** Descriptive statistics of patient-centered variables of questions 8 and 9 after 28 days in the three groups and the results of the significance testing. SD: standard deviation; TBs: traditional brackets; SLBs: self-ligating brackets; SLBs+P: self-ligating brackets + piezocision; N: number of patients; NS: There was no statistically significant difference at P > 0.05. †Employing Fisher’s Exact Test

		TBs		SLBs		SLBs+P		P-value†	Significance
		Yes	No	Yes	No	Yes	No		
Q8: Friend recommendation	N (%)	20 (100)	0 (0)	20 (100)	0 (0)	19 (95)	1 (5)	0.655	NS
Q9: Repeating the procedure	N (%)	20 (100)	0 (0)	20 (100)	0 (0)	19 (95)	1 (5)	0.655	NS

Posthoc tests regarding the previous variables at T1, T2, and T3 showed that the differences were mainly between the SLBs+P group and the other two groups, where the mean values were greater in the SLBs+P group. In contrast, there were no significant statistical differences between the SLBs alone and TBs alone (Tables 6-7). Concerning patients’ satisfaction with the provided treatment, a statistically significant difference between the three groups was detected after 28 days (P < 0.001). The SLBs+P group showed the lowest mean values, whereas there were no significant statistical differences between the two other groups. All patients in both SLB alone and TB alone groups answered that they would accept undergoing this treatment again and would advise a friend to receive such procedures, while 95% answered yes to the last two questions in the SLBs+P group.

**Table 6:** Posthoc tests for pairwise comparisons concerning questions 1 and 2 of the first questionnaire. T1: after 24 hours of the beginning of orthodontic treatment; T2: after 4 days; T3: after 7 days; TBs: traditional brackets; SLBs: self-ligating brackets, SLBs+P: self-ligating brackets + piezocision; NS: There was no statistically significant difference at P > 0.05 †Bonferroni was used to detect any significant difference between the two groups *statistically significant at P < 0.001 **statistically significant at P < 0.01 ***statistically significant at P < 0.05

		Groups	Mean difference	95% CI for difference		P-value^†^	Significance
				Lower bound	Upper bound		
Q1 Pain	T1	TBs vs. SLBs	1.42	-4.51	7.36	1.000	NS
		TBs vs. SLBs+P	18.72	-24.66	-12.79	>0.001	*
		SLBs vs. SLBs+P	- 20.15	-26.17	-14.15	>0.001	*
	T2	TBs vs. SLBs	0.68	-5.15	6.53	1.000	NS
		TBs vs. SLBs+P	-7.36	-13.21	-1.53	0.009	**
		SLBs vs. SLBs+P	-8.05	-13.97	-2.14	0.004	**
	T3	TBs vs. SLBs	-0.20	-4.36	3.95	1.000	NS
		TBs vs. SLBs+P	-9.88	-14.04	-5.74	>0.001	*
		SLBs vs. SLBs+P	-9.68	-13.89	-5.48	>0.001	*
Q2 Discomfort	T1	TBs vs. SLBs	1.26	-4.48	7.02	1.000	NS
		TBs vs. SLBs+P	-17.52	-23.27	-11.77	>0.001	*
		SLBs vs. SLBs+P	-18.78	-24.61	-12.97	>0.001	*
	T2	TBs vs. SLBs	9.68	1.62	17.74	0.013	***
		TBs vs. SLBs+P	-3.95	-12.01	4.11	0.695	NS
		SLBs vs. SLBs+P	-13.63	-21.80	-5.47	>0.001	*
	T3	TBs vs. SLBs	8.02	3.43	12.62	>0.001	*
		TBs vs. SLBs+P	-6.02	-10.62	-1.43	0.005	**
		SLBs vs. SLBs+P	-14.05	-18.70	-9.40	>0.001	*

**Table 7:** Post-hoc tests for pairwise comparisons of questions 3, 4, 5, 6, and 7. T1: after 24 hours of the beginning of orthodontic treatment; T2: after 4 days; T3: after 7 days; TBs: traditional brackets; SLBs: self-ligating brackets; SLBs+P: self-ligating brackets + piezocision; NS: There was no statistically significant difference at P > 0.05. †Mann-Whitney U test was used to detect any significant difference between the two groups ‡Significance level was adjusted according to Bonferroni correction *statistically significant at P < 0.001 **statistically significant at P < 0.017

		Groups	Mean Rank	P-value^†^	Significance ‡	
						Q3 Swelling	T1	TBs vs. SLBs	19.98	0.971	NS	
	TBs vs. SLBs+P	20.03	>0.001	*		
	SLBs vs. SLBs+P	10.50	>0.001	*		
T2	TBs vs. SLBs	30.00	0.971	NS		
	TBs vs. SLBs+P	10.00	>0.001	*		
	SLBs vs. SLBs+P	29.00	>0.001	*		
T3	TBs vs. SLBs	19.98	0.971	NS		
	TBs vs. SLBs+P	20.03	0.001	**		
	SLBs vs. SLBs+P	10.50	0.001	**		
Q4 Mastication	T1	TBs vs. SLBs	19.93	0.966	NS	
		TBs vs. SLBs+P	20.03	>0.001	*	
		SLBs vs. SLBs+P	11.50	>0.001	*	
	T2	TBs vs. SLBs	28.95	0.002	**	
		TBs vs. SLBs+P	11.00	0.001	**	
		SLBs vs. SLBs+P	28.00	>0.001	*	
	T3	TBs vs. SLBs	25.48	0.269	NS	
		TBs vs. SLBs+P	14.24	0.001	**	
		SLBs vs. SLBs+P	14.15	0.001	**	
Q5 Swallowing	T1	TBs vs. SLBs	19.98	0.985	NS	
		TBs vs. SLBs+P	20.03	>0.001	*	
		SLBs vs. SLBs+P	12.85	>0.001	*	
	T2	TBs vs. SLBs	27.53	0.853	NS	
		TBs vs. SLBs+P	12.24	0.001	**	
		SLBs vs. SLBs+P	26.76	0.001	**	
	T3	TBs vs. SLBs	20.33	0.971	NS	
		TBs vs. SLBs+P	19.66	>0.001	*	
		SLBs vs. SLBs+P	14.03	>0.001	*	
Q6 Jaw movement restriction	T1	TBs vs. SLBs	21.15	0.467	NS	
		TBs vs. SLBs+P	18.79	>0.001	*	
		SLBs vs. SLBs+P	10.50	>0.001	*	
	T2	TBs vs. SLBs	30.00	0.590	NS	
		TBs vs. SLBs+P	10.00	>0.001	*	
		SLBs vs. SLBs+P	29.00	>0.001	*	
	T3	TBs vs. SLBs	20.85	1.000	NS	
		TBs vs. SLBs+P	19.11	>0.001	*	
		SLBs vs. SLBs+P	11.60	>0.001	*	
Q7 Satisfaction	T3	TBs vs. SLBs	19.40	0.671	NS	
		TBs vs. SLBs+P	20.63	0.002	**	
		SLBs vs. SLBs+P	25.35	>0.001	*	

## Discussion

This is the first RCT assessing the patient-centered outcomes associated with the use of piezocision in combination with SLBs compared to the solitary use of TBs or SLBs in correcting severe crowding of upper anterior teeth in adult patients who underwent extraction-based orthodontic treatment. The VAS was used in this study because it is considered a simple, applicable, and valid tool for assessing pain and other patient-centered outcomes [28-30]. It is also one of the most popular scales that has been used in a large number of previous studies [18,23,27]. No measurement was taken before 24 hours of the beginning of treatment to avoid the local anesthesia effect in the SLBs+P group.

The mean levels of pain and discomfort in the SLBs+P group were greater than those in the other two groups after 1 day, 4 days, and 7 days, with statistically significant differences (P < 0.001). After that, the pain and discomfort began to decrease in the three groups until they reached their lowest levels after 28 days. The greater perception of pain and discomfort in the SLBs+P group may be attributed to the effect of surgical intervention applied to the patients of this group compared to the other two groups.

The maximum pain levels in the SLBs+P group were 24 hours after the surgical intervention, then they declined significantly after 7 days, reaching their lowest values after 28 days. This can be explained by the surgical protocol followed in this study, which depended on the use of blade No. 15 first, followed by the BS1 piezoelectric tip. This procedure allowed the piezoelectric force to be delivered directly to the hard tissues, protecting soft tissues and periosteum from rupture. In addition, the chosen micro-saw was designed to make safe and selective incisions, maintaining the roots’ integrity and resulting in less postsurgical pain.

The swelling was reported in the SLBs+P group only, which was at its greatest level after 24 hours of surgical intervention, then decreased significantly after 7 days, to become almost absent after 28 days. After reviewing the literature, we found only one study conducted by Gibreal and his colleagues, which investigated the swelling associated with the piezocision. The current results agree with those of Gabriel’s study, which showed that the greatest mean values of swelling were after the first 24 hours and were within the acceptable values, then gradually decreased within a week of the surgical intervention. This may be attributed to the minimally invasive nature of piezocision [16].

The levels of mastication and swallowing difficulties were at the greatest levels after 24 hours of orthodontic treatment in the SLBs+P group and decreased after that. The mastication and swallowing difficulties can be explained by the orthodontic forces applied at the beginning of the treatment, which were new forces that the patient did not adapt to yet, in addition to the effects of the surgical intervention and the accompanying edema during the first days.

The results of our study are consistent with those of Gibreal and his colleagues’ trial, which is the only study that investigated mastication and swallowing difficulties associated with the piezocision, where those difficulties were at their highest levels after 24 hours and gradually decreased during the first week [16].

This study showed that the levels of satisfaction were very high in both TBs alone and SLBs alone groups, where the average values were 98.30 and 98.95, respectively. The satisfaction levels were also great in the SLBs with the piezocision group (91.89) but slightly lower than in the other two groups. The high levels of satisfaction with the surgical procedures can be justified by the conservative nature of the minimally invasive corticotomy, which did not require elevating flaps or suturing in addition to the less traumatic and painful cuts in comparison with the traditional surgical techniques. Therefore, the discomfort and pain were minimal, and the need to take analgesics was low. The current results agree with previous studies where the satisfaction levels in the experimental group using piezocision were high [9,16].

Most patients in the SLBs+P group (95%) reported that they would experience the therapeutic procedures again and that they would advise a friend. From the same perspective in Charavet et al. study, patients showed their willingness to undergo the treatment procedure again and recommend friends to undergo a similar surgical intervention [9]. There were no notable harms during the period of this trial. Participants did not report any serious side effects or postsurgical complications (i.e., edema, hematomas, numbness, gingival recession).

Limitations

The current RCT has some limitations. First, the Hawthorne effect and detection bias are possible to happen due to the inability to blind both patients and the principal researcher. Secondly, long-term complications following corticotomy intervention, such as root resorption, scars, and the vitality of teeth, are important variables to be evaluated in similar future studies. Thirdly, the current trial did not assess the periodontal indices (e.g., gingival recession, alveolar bone) at the end of the treatment. Finally, mensuration of patient-centered outcomes using other acceleration methods in different malocclusion cases is recommended in forthcoming research.

## Conclusions

The levels of pain and discomfort, swelling, difficulties in mastication and swallowing, and restriction of jaw movement were greater in SLBs with the piezocision group compared with the solitary use of SLBs or TBs in the first week only. The patients showed a high level of satisfaction with the therapeutic procedures applied, which means that SLBs or piezocision can be used alone or together as acceptable methods by patients to accelerate the orthodontic movement.
